# The Metastasis Pattern of Renal Cell Carcinoma Is Influenced by Histologic Subtype, Grade, and Sarcomatoid Differentiation

**DOI:** 10.3390/medicina59101845

**Published:** 2023-10-17

**Authors:** Hyung Kyu Park

**Affiliations:** Department of Pathology, Chungnam National University School of Medicine, Daejeon 35015, Republic of Korea; hkpark@g.cnu.ac.kr

**Keywords:** renal cell carcinoma, clear-cell renal carcinoma, chromophobe renal cell carcinoma, papillary renal cell carcinoma, metastasis

## Abstract

*Background and Objectives*: Metastasis is a major cause of death in renal cell carcinoma (RCC) patients; therefore, a better understanding of the metastatic process and the ability to predict metastasis in advance is important for treating patients with RCC. This study aimed to investigate whether histological subtypes of RCC and other factors, such as nuclear grade and sarcomatoid differentiation, could predict the probability and location of metastases in patients with RCC. *Materials and Methods*: Cases of clear-cell, papillary, chromophobe, and sarcomatoid RCC were retrieved and analyzed from the Surveillance, Epidemiology, and End Results databases. *Results*: When comparing the metastatic patterns among the three histologic subtypes, patients with clear-cell RCC were significantly more likely to have brain and lung metastases. Moreover, patients with papillary RCC were significantly less likely to develop bone metastases and more likely to develop lymph node metastases. Patients with chromophobe RCC are significantly more likely to develop liver metastases. As the nuclear grade increased, there was also a significantly increased tendency for clear-cell RCC to metastasize to the lungs. Patients with sarcomatoid RCC had a higher rate of metastasis, with a significantly higher probability of metastasis to the bone and lungs, than those with all three histological subtypes did. *Conclusions*: Histological subtype, nuclear grade, and sarcomatoid differentiation were significant predictors of metastasis in patients with RCC.

## 1. Introduction

According to the GLOBOCAN 2020 database, kidney cancer accounts for approximately 2.2% of newly diagnosed cancers and 1.8% of cancer-related deaths worldwide, and its incidence is expected to increase in the future [1,2]. Renal cell carcinoma (RCC) is the most common type of primary kidney carcinoma, accounting for approximately 90% of all primary kidney malignancies [3,4], and refers to a diverse group of tumors that exhibit unique differences in their histological, molecular, clinical outcomes, and responses to therapy [5]. Notably, more than 17 RCC subtypes were recognized in the fifth edition of the WHO Classification of Urinary and Male Genital Tumors [6,7]. However, three major subtypes account for approximately 90% of all RCC cases: clear-cell RCC, which accounts for 60–75% of cases; papillary RCC, which accounts for 13–20% of cases; and chromophobe RCC, which accounts for 5–7% of all RCCs [7,8,9].

Several studies have shown that different histological subtypes of RCC have different clinical presentations and prognoses in several studies [5,7,10,11,12]. In addition to histologic subtypes, numerous studies have been conducted to predict the prognosis of RCC but the most commonly used are Fuhrman nuclear grades, World Health Organization/International Society of Urological Pathology (WHO/ISUP) nuclear grades, and the presence or absence of sarcomatous differentiation [13,14,15,16,17,18]. Much research has been conducted to predict the prognosis of renal cancer, including attempts to use genetic analysis and artificial intelligence to predict the prognosis [5,19,20].

The presence or absence of metastasis is one of the most important factors in determining the prognosis of patients with RCC. While the overall 5-year relative survival rate for patients with kidney and renal pelvic cancers is 77.6%, the 5-year relative survival rate for patients with metastases is only 17.4%; in contrast, patients with localized disease had a 5-year relative survival rate of 92.9% [21]. Considering that approximately 15% of patients have metastatic disease at the time of diagnosis, a deeper understanding of the pathophysiology of metastatic RCC is important for improved treatment [21,22,23,24,25].

Metastasis is a complex process mediated by various factors and is poorly understood. Therefore, many researchers are still investigating this process, and recent studies have reported that different histological tumor subtypes have different metastatic patterns [26,27,28]. The association between histologic subtypes and metastatic patterns has also been reported in studies involving RCC patients [11,12].

Prior studies have focused solely on examining the association between metastatic behavior and histological subtypes; however, they did not investigate whether histological subtypes were the only factors influencing metastatic behavior in patients with RCC. Other factors, such as nuclear grade and sarcomatous differentiation, have been recognized to impact the prognosis of patients with RCC; however, it is not known if they have any effect on metastatic behavior.

This study aimed to verify that RCC shows a unique pattern of metastasis depending on the histologic subtype, as previously found, and to determine whether other prognostic factors beyond the histologic subtype, such as nuclear grade and sarcomatous differentiation, also affect the metastatic pattern of RCC. A database provided by the Surveillance, Epidemiology, and End Results (SEER) program was examined.

## 2. Materials and Methods

### 2.1. Data Selection

The SEER database was accessed using the Case Listing Session of SEER*Stat software version 8.4.1.2 [29]. Among the databases provided by the SEER program, the “Incidence—SEER Research Data, 17 Registries, Nov 2022 Sub (2000–2020)” database was selected [30]. The selected database was searched for patients who met the following inclusion criteria: (1) Having received a diagnosis in 2010 or later. The SEER program began recording the presence or absence of metastases to the bone, brain, liver, and lungs detected at the time of diagnosis in 2010. Therefore, this study included only patients diagnosed in 2010 or later; (2) Malignant primary kidney neoplasms. The International Classification of Diseases for Oncology (ICD-O-3) anatomical site codes were used to define the primary sites; (3) patients were diagnosed with clear-cell RCC, papillary RCC, chromophobe RCC, and sarcomatoid RCC. The ICD-O-3 histology codes were used to select patients for each diagnosis. Specifically, code 8310/3 was used to select patients with clear-cell RCC, and code 8260/3 was used to select patients with papillary RCC; furthermore, codes 8270/3 and 8317/3 were used to select patients with chromophobe RCC, and code 8318/3 was used to select patients with sarcomatoid RCC.

For each selected patient, the extracted data comprised the year of diagnosis; ICD-O-3 histology codes; Fuhrman nuclear grade (for patients diagnosed in 2017); WHO/ISUP nuclear grade (for patients diagnosed in 2018 and later); M stage; and information on specific metastatic sites, including the bone, brain, liver, lung, distant lymph nodes (LNs), and other organs. Information regarding the presence or absence of metastases to distant LNs and other organs was available only for patients diagnosed in 2016 or later.

### 2.2. Definition of Organotropic Metastasis Rate

Traditionally, the metastasis rate—which is the percentage of patients with metastases among the total number of patients—is used to measure and compare the tendency of tumors to metastasize; however, using this method of calculating the overall metastasis rate to measure and compare the tendency of tumors to metastasize to specific organs is not appropriate and may lead to statistical errors. This is because if the method for calculating the overall metastasis rate and the method for calculating the metastasis rate to specific organs were the same, tumors with a high overall metastasis rate would have a high metastasis rate to specific organs, and conversely, tumors with a low overall metastasis rate would have a low metastasis rate to specific organs. Consequently, it becomes challenging to accurately compare the tendency for organ-specific metastasis among different tumors with significantly different overall metastasis rates. Therefore, to accurately compare the tendency of metastasis to a specific organ, the statistical bias caused by differences in overall metastasis rates must be corrected. Instead of using the entire patient population, the percentage of patients with metastasis to a specific organ among those who presented with any form of metastasis was calculated and used for comparison. This methodology was used in previous studies [26,27,28]. For clarity, this value is described as the organotropic metastasis rate.

### 2.3. Statistical Analysis

Pearson’s chi-square test or Fisher’s exact test was used to compare categorical variables, such as the presence of metastases, as appropriate. Statistical analyses were performed using SPSS software (version 17.0; SPSS Inc., Chicago, IL, USA) and OriginPro (version 2023b; OriginLab, Northampton, MA, USA).

## 3. Results

### 3.1. Histologic Subtypes

A total of 83,558 patients with clear-cell RCC, 17,638 patients with papillary RCC, and 7847 patients with chromophobe RCC were identified. The overall metastasis rate (percentage of patients with metastasis/total number of patients) of clear-cell RCC patients was 10.2% (8549/83,558), which was significantly (both *p* < 0.001) higher than that of papillary RCC patients (4.6%, 808/17,638) and chromophobe RCC patients (1.9%, 146/7847). There was also a significant (*p* < 0.001) difference between the overall metastasis rates of papillary RCC patients and that of chromophobe RCC patients.

Because of the substantial differences in the overall metastasis rates observed between the three histological subtypes, direct comparisons of the metastatic rates to specific organs between these three histological types are likely to be statistically inaccurate; therefore, organotropic metastasis rates (the percentage of patients with metastasis to the indicated organ/total number of patients with metastasis) were calculated as described in the Methods section and used to compare the metastatic tendency to specific organs among different histologic subtypes.

**Figure 1:**
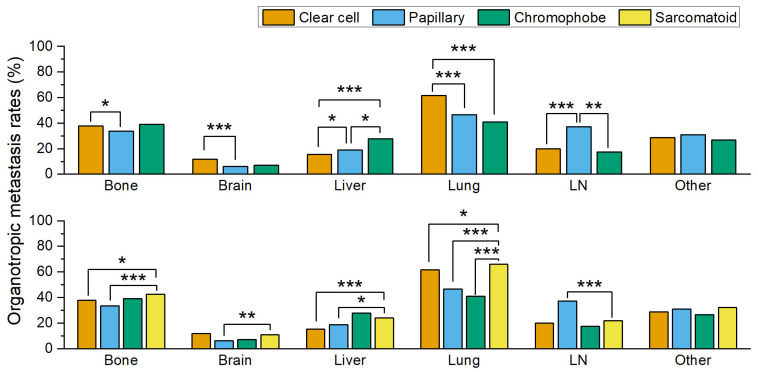
Comparison of organotropic metastasis rates among different histologic subtypes of renal cell carcinoma. Only statistically significant comparisons are marked with asterisks (*, *p* ≤ 0.05; **, *p* ≤ 0.01; ***, *p* ≤ 0.001).

The calculated and compared organotropic metastasis rates are summarized in Table 1 and Figure 1. The bone organotropic metastasis rate in patients with papillary RCC (33.6%, 267/795) was significantly (*p* = 0.022) lower than that in patients with clear-cell RCC (37.7%, 3173/8417). The brain organotropic metastasis rate in patients with clear-cell RCC (11.9%, 996/8375) was significantly (*p* < 0.001) higher than that in patients with papillary RCC (6.1%, 48/790). The liver organotropic metastasis rate in patients with chromophobe RCC (27.6%, 40/145) was significantly (*p* < 0.001 and *p* = 0.015) higher than that in patients with clear-cell RCC (15.3%, 1281/8383) and patients with papillary RCC (18.8%, 148/789). The liver organotropic metastasis rate in patients with papillary RCC was also significantly (*p* = 0.01) higher than that in patients with clear-cell RCC. Furthermore, the lung organotropic metastasis rate in patients with clear-cell RCC (61.6%, 5156/8368) was significantly (both *p* < 0.001) higher than that in patients with papillary RCC (46.6%, 368/789) and patients with chromophobe RCC (41.0%, 59/144). The LN organotropic metastasis rate in patients with papillary RCC (37.2%, 146/393) was significantly (*p* < 0.001 and *p* = 0.001) higher than that in patients with clear-cell RCC (19.8%, 846/4279) and patients with chromophobe RCC (17.5%, 14/80). Finally, organotropic metastasis rates to other organs showed no significant differences among patients with clear-cell RCC (28.7%, 1234/4298), papillary RCC (30.9%, 121/391), and chromophobe RCC (26.6%, 21/79).

In summary, patients with clear-cell RCC had significantly higher brain and lung organotropic metastasis rates. Moreover, patients with papillary RCC had significantly lower bone organotropic metastasis rates and significantly higher LN organotropic metastasis rates. Finally, patients with chromophobe RCC had significantly higher rates of liver organotropic metastasis.

### 3.2. Sarcomatoid Differentiation

In total, 1187 patients with sarcomatoid RCC were identified and retrieved. The overall metastasis rate in patients with sarcomatoid RCC (56.7%, 673/1187) was significantly (all *p* < 0.001) higher than that in patients with clear-cell RCC (10.2%, 8549/83,558), patients with papillary RCC (4.6%, 808/17,638), and patients with chromophobe RCC (1.9%, 146/7847).

The calculated and compared organotropic metastasis rates are summarized in Table 1 and Figure 1. Interestingly, patients with sarcomatoid RCC showed unique patterns of organotropic metastases. The bone organotropic metastasis rate was significantly (*p* = 0.015 and *p* = 0.001) higher in patients with sarcomatoid RCC (42.4%, 281/662) than in those with clear-cell RCC (37.7%, 3173/8417) and those with papillary RCC (33.6%, 267/795). Similarly, the lung organotropic metastasis rate was significantly (*p* = 0.026, *p* < 0.001 and *p* < 0.001) higher in patients with sarcomatoid RCC (66.0%, 433/656) than in those with clear-cell RCC (61.6%, 5156/8368), those with papillary RCC (46.6%, 368/789), and those with chromophobe RCC (41.0%, 59/144).

The brain organotropic metastasis rate in patients with sarcomatoid RCC was 11.0% (72/657), which was significantly higher (*p* = 0.001) than the 6.1% rate (48/790) found in patients with papillary RCC. Conversely, the brain organotropic metastasis rate of clear-cell RCC (11.9%, 996/8375) showed no significant difference (*p* = 0.475) when compared to sarcomatoid RCC. Similarly, the liver organotropic metastasis rate in patients with sarcomatoid RCC (23.9%, 156/654) was significantly (*p* < 0.001 and *p* = 0.018) higher than that in patients with clear-cell RCC (15.3%, 1281/8383) and patients with papillary RCC (18.8%, 148/789); moreover, when compared to the organotropic metastasis rate in patients with chromophobe RCC (27.6%, 40/145), there was no significant difference (*p* = 0.345).

In contrast, the LN organotropic metastasis rate in patients with sarcomatoid RCC (21.9%, 44/201) was significantly (*p* < 0.001) lower than that in papillary RCC (37.2%, 146/393), and there were no significant (*p* = 0.462 and *p* = 0.412) differences in the LN organotropic metastasis rates in patients with clear-cell RCC (19.8%, 846/4279) and patients with chromophobe RCC (17.5%, 14/80). The rate of organotropic metastasis to other organs in patients with sarcomatoid RCC (32.2%, 65/202) was not significantly (*p* = 0.288, *p* = 0.759, and *p* = 0.360) different from that in patients with clear-cell RCC (28.7%, 1234/4298), patients with papillary RCC (30.9%, 121/391), and patients with chromophobe RCC (26.6%, 21/79).

In summary, the bone and lung organotropic metastasis rates of sarcomatoid RCC patients were significantly higher than those of the three common histologic types (clear-cell, papillary, and chromophobe RCC), whereas the brain and liver organotropic metastasis rates were similar to the highest among the other three histologic types (clear-cell RCC in the brain and chromophobe RCC in the liver) and higher than those of the other two histologic types. The LN organotropic metastasis rate in patients with sarcomatoid RCC was significantly lower than that in patients with papillary RCC and similar to the other two histologic subtypes. The rate of organotropic metastasis to other organs in patients with sarcomatoid RCC was not significantly different from that in patients with the other three histologic subtypes.

### 3.3. Nuclear Grade

Fuhrman nuclear grade data were available for 48,704 of the 83,558 patients diagnosed with clear-cell RCC. While Fuhrman nuclear grade data were also available for patients with papillary RCC, we restricted our analysis to patients with clear-cell RCC to simplify the statistical analysis.

Of the 48,704 patients with clear-cell RCC, 5652 were classified as grade 1, 26,137 were classified as grade 2, 13,495 were classified as grade 3, and 3420 were classified as grade 4. Patients diagnosed with Fuhrman grade 1 clear-cell RCC displayed a metastasis rate of 2.2% (126 out of 5652 cases). Those with Fuhrman grade 2 clear-cell RCC experienced a slightly elevated metastasis rate of 3.5% (907 out of 26,137 cases). In cases where the diagnosis indicated Fuhrman grade 3 clear-cell RCC, the metastasis rate notably increased to 11.0% (1478 out of 13,495 cases). The most substantial metastasis rate was observed in patients with Fuhrman grade 4 clear-cell RCC, reaching 30.2% (1034 out of 3420 cases). When the metastasis rates for each grade were compared, there was a clear upward trend with increasing grades, with statistically significant differences observed between grades (all *p* < 0.001).

**Figure 2:**
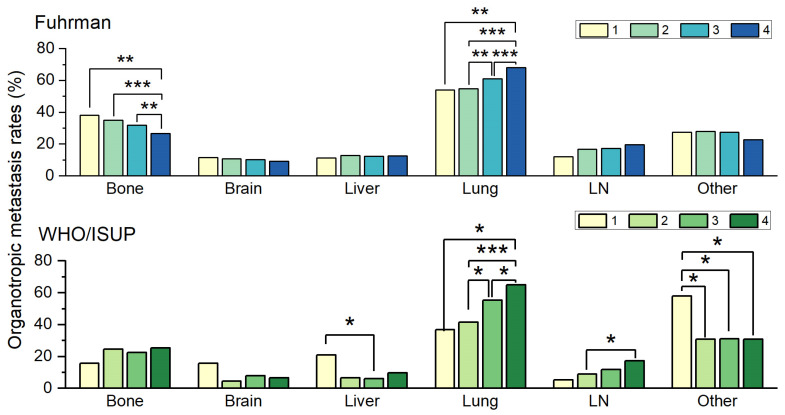
The relationship between changes in organotropic metastasis rates and changes in Fuhrman nuclear grade and World Health Organization/International Society of Urological Pathology (WHO/ISUP) grade. Only statistically significant comparisons are marked with asterisks (*, *p* ≤ 0.05; **, *p* ≤ 0.01; ***, *p* ≤ 0.001).

The calculated organotropic metastasis rates in patients with each Fuhrman grade and their comparisons are shown in Table 2 and Figure 2. Interestingly, the lung organotropic metastasis rate increased with the nuclear grade. Although there was no significant difference (*p* = 0.892) between the lung organotropic metastasis rate of patients with Fuhrman grade 1 clear-cell RCC (54.1%, 66/122) and Fuhrman grade 2 clear-cell RCC (54.7%, 490/895), statistically significant (*p* = 0.003 and *p* < 0.001) differences were observed between the lung organotropic metastasis rate of patients with Fuhrman grade 2 clear-cell RCC and that of Fuhrman grade 3 clear-cell RCC (61.0%, 891/1460) patients, as well as between the lung organotropic metastasis rate of patients with Fuhrman grade 3 clear-cell RCC and that of those with Fuhrman grade 4 clear-cell RCC (68.1%, 694/1019). The LN organotropic metastasis rate also increased with increasing nuclear grade; however, the degree of increase was insufficient to reach statistical significance. Moreover, the bone organotropic metastasis rate in patients with Fuhrman grade 4 clear-cell RCC (26.5%, 272/1028) was significantly (*p* = 0.006, *p* < 0.001, and *p* = 0.004, respectively) lower than that in patients with Fuhrman grades 1 (38.2%, 47/123), 2 (34.9%, 313/898), and 3 (31.8%, 463/1457). Finally, the rates of organotropic metastasis to the brain, liver, and other organs did not show significant differences among grades.

WHO/ISUP nuclear grade data were available for 19,351 of the 83,558 patients diagnosed with clear-cell RCC. Similar to the previous analysis of the Fuhrman nuclear grade, only patients diagnosed with clear-cell RCC were analyzed to simplify the statistical analysis.

Of the 19,351 patients with clear-cell RCC, 2311 were classified as WHO/ISUP nuclear grade 1; 10,232 patients were classified as grade 2; 4967 patients were classified as grade 3; and 1841 patients were classified as grade 4. The overall metastatic rates in patients with clear-cell RCC, graded according to the WHO/ISUP grades, were 0.8% (19/2311), 1.3% (136/10,232), 5.9% (295/4967), and 22.6% (416/1841) for grades 1, 2, 3, and 4, respectively. Similar to the Fuhrman nuclear grade, clear-cell RCC patients divided into WHO/ISUP nuclear grades also showed an increasing overall metastasis rate with increasing grade, and the difference in the metastasis rate between each grade was statistically significant (*p* = 0.046 between grades 1 and 2 and *p* < 0.001 for all others).

The calculated organotropic metastasis rates in patients with each WHO/ISUP nuclear grade and their comparisons are shown in Table 2 and Figure 2. The analysis of the difference in the organotropic metastasis rate between the WHO/ISUP nuclear grades revealed some similarities to the previous analysis of changes in the organotropic metastasis rate in Fuhrman nuclear grades but there were also some differences.

Further, the rate of lung organotropic metastasis increased with increasing WHO/ISUP grades, similar to when analyzing the Fuhrman grade. There was also no statistically significant (*p* = 0.708) difference in the lung organotropic metastasis rate between grades 1 (36.8%, 7/19) and 2 (41.4%, 55/133) but a significant (*p* = 0.008 and *p* = 0.01, respectively) difference in the metastasis rate between grades 2 and 3 (55.3%, 163/295) and grades 3 and 4 (64.9%, 268/413) was observed, similar to the analysis of the Fuhrman grade. Similar to the Fuhrman grade analysis, the LN organotropic metastasis rate also increased with grade; however, this correlation was not statistically significant, similar to a previous analysis of the Fuhrman grade. In contrast to the previous Fuhrman grade analysis, there was no significant difference in the bone organotropic metastasis rate according to WHO/ISUP grade. Additionally, the organotropic metastasis rate to other organs in patients classified as grade 1 (57.9%, 11/19) was significantly (*p* = 0.036, *p* = 0.016, *p* = 0.014, respectively) higher than that in patients classified as grade 2 (30.9%, 42/136), grade 3 (31.2%, 92/295), or grade 4 (30.9%, 128/414). No statistically significant differences were observed in organotropic metastasis rates in the brain and liver by grade.

## 4. Discussion

Metastasis is the major cause of death in patients with advanced RCC [3]; thus, an improved understanding of the metastatic process is essential for providing better care for patients with advanced RCC. This study examined the impact of the histologic subtype, Fuhrman and WHO/ISUP nuclear grades, and the presence of sarcomatoid differentiation on the metastatic behavior of RCC based on a large population-based database provided by the SEER program. All three factors analyzed in this study, namely histologic subtype, Fuhrman and WHO/ISUP nuclear grade, and sarcomatoid differentiation, were analyzed during pathological examination. Pathologists can easily assess all three factors through microscopic observation and are advised to document them in pathology reports, especially for resection specimens of RCC [4]. Therefore, the outcomes of this study are expected to support clinicians in determining a monitoring plan for patients with RCC without requiring additional steps.

This study analyzed only three histologic subtypes, clear-cell RCC, papillary RCC, and chromophobe RCC, despite the fifth edition of the WHO classification documenting more than seventeen subtypes [7]. Previously, sarcomatous differentiation was considered a distinct histological subtype; however, it is now recognized as a feature that may be present across histological subtypes and is not classified as a distinct subtype [13,31]. Several other histological subtypes were not included in this study because the three histological subtypes analyzed in this study (clear cell, papillary, and chromophobe) are observed in approximately 90% of patients with kidney cancer. The remaining histological subtypes are much less common and were classified relatively recently. As a result, either they are not recorded as distinct subtypes in the SEER database, or if they are, their quantities are insufficient for meaningful statistical analysis. Our statistical methodology for comparing metastatic patterns required a sufficient number of patients with metastases, and it was challenging to collect sufficient cases, even for the three relatively common histological subtypes. In particular, chromophobe RCC, the rarest of the three histological subtypes included in this study, had the lowest rate of metastasis (1.9%, 146/7847), and we were only able to identify 146 cases with metastasis. This limits our ability to statistically compare chromophobe RCC with the other two subtypes in this study and makes it difficult to demonstrate a statistically significant difference. Nevertheless, the results of this study are sufficient to validate those of previous studies.

The high tendency of clear-cell RCC to metastasize to the lungs and brain, of papillary RCC to metastasize to distant LNs, and of chromophobe RCC to metastasize to the liver observed in this study is consistent with previous studies [11]. Here, we also observed a lower bone organotropic metastasis rate in papillary RCC, which has not been previously reported. However, the difference between the bone organotropic metastasis rates of papillary RCC (33.6%, 267/795) and clear-cell RCC (37.7%, 3173/8417), although statistically significant (*p* = 0.022), was not that huge. Whether this difference is a temporary finding in this study or a clinically important finding that could be confirmed in other studies is a matter for future research.

Interestingly, chromophobe RCC demonstrated a higher tendency for liver metastasis. A higher tendency to metastasize to the liver has been reported as a characteristic of neuroendocrine neoplasms in previous studies on tumors arising from other organs such as the lungs, stomach, and colon [26,27,28]. Unfortunately, neuroendocrine neoplasms are rare in the kidney; therefore, we could not compare the metastatic behaviors of neuroendocrine neoplasms and chromophobe RCC. However, it is interesting to note that two different tumors, neuroendocrine neoplasms and chromophobe RCC, exhibit similar metastatic behaviors. Further studies analyzing the common features of these two tumors may improve our understanding of the metastatic process.

In addition to the metastatic tendencies observed in this study, a previous study has reported that clear-cell RCC has a high tendency to metastasize to the adrenal gland and pancreas, whereas papillary RCC has a high tendency to metastasize to the peritoneum [11]. Unfortunately, these findings were not confirmed in the present study. This study was based on the SEER database, which categorizes metastases to the bone, brain, liver, lungs, lymph nodes, and other organs when collecting metastasis records. Metastases to the adrenal gland, pancreas, and peritoneum are lumped together as metastases to other organs in the SEER database and cannot be analyzed separately, representing a limitation of the present study. However, RCC metastases most commonly go to the lungs, lymph nodes, bones, liver, adrenal glands, and brain. This study analyzed metastases to all organs except the adrenal glands [11]. Therefore, this study is of sufficient importance.

The overall metastasis rate of sarcomatoid RCC was 56.7% (673/1187), which was significantly higher than that of clear-cell RCC (10.2%), papillary RCC (4.6%), and chromophobe RCC (1.9%). This is approximately five times higher than the overall metastasis rate of clear-cell RCC, which has the highest metastasis rate among the three histological subtypes of RCC. Although this metastasis rate was lower than that reported in a previous study (66.2%, 51/77) [13], it is understandable that the SEER database used in this study only recorded metastases detected at the time of diagnosis. This high metastasis rate is likely one of the reasons for the poor prognosis associated with sarcomatoid RCC [31,32].

Sarcomatoid RCC demonstrates a distinct organotropic metastasis pattern, featuring a notably higher tendency for bone and lung metastases than that of the three common histologic subtypes. And its tendency for organotropic metastasis to the brain and liver was similar to the highest among the three common histologic subtypes. In contrast, the tendency of organotropic metastasis to the lymph nodes was similar to that of clear-cell RCC or chromophobe RCC and was significantly lower than that of papillary RCC. The tendency of organotropic metastasis to other organs was not significantly different among the three common histologic subtypes. Comparing these unique metastatic behaviors to those of the three common histologic subtypes, they appear similar to the patterns of clear-cell RCC with a high tendency for brain and lung metastasis and chromophobe RCC with high liver metastasis tendencies. Interestingly, a high tendency for lymph node metastasis—characteristic of papillary RCC—was not observed, which may be related to the fact that papillary RCC is the least likely of the three histological subtypes to exhibit sarcomatoid differentiation [13].

The relationship between the nuclear grade and metastatic behavior was also analyzed. Fuhrman nuclear grades have been widely used since they were first reported in 1982 [33]. The WHO/ISUP nuclear grade was introduced in 2012 by the ISUP 2012 Consensus Conference to replace the Fuhrman nuclear grade [16]. Since its acceptance in the WHO classification in 2016, it has been used as a replacement for the Fuhrman nuclear grade [8,15]. It could be argued that it would be sufficient to analyze only the WHO/ISUP nuclear grades that are currently in use; however, the SEER database collected Fuhrman nuclear grade information until 2017 and began collecting WHO/ISUP nuclear grade information in 2018. Consequently, there were more patients with Fuhrman nuclear grade information than there were with WHO/ISUP nuclear grade information among those diagnosed between 2010 and 2020 included in this study. Furthermore, the WHO/ISUP nuclear grading system represents an improvement over the Fuhrman nuclear grading system rather than a completely new one. Therefore, the correlation between the Fuhrman nuclear grade and metastatic behavior may be useful as an adjunct to the information provided by the correlation between the WHO/ISUP nuclear grade and metastatic behavior.

The trend of increasing lung metastases with increasing nuclear grade was the only statistically significant finding common to both the Fuhrman and WHO/ISUP nuclear grades. Two possible interpretations can be made based on this finding. First, as the lung is the organ to which RCC most commonly metastasizes, changes in all other metastatic patterns may be particularly notable in lung metastases. The second interpretation is that the tendency to metastasize to the lungs may increase as nuclear grade increases. If this were the case, it would be an interesting finding.

This study demonstrated that there are significant differences in the metastatic behavior of RCCs depending on the histologic subtype, sarcomatous differentiation, and nuclear grade. Previous studies also have reported various factors that may influence the metastatic behavior of RCC. For example, it has been reported that clear-cell RCCs have different metastatic potential and prognosis depending on their genetic characteristics, especially the degree of intra-tumor heterogeneity and somatic copy number alterations [34]. And many studies have also shown genetic differences between the primary and metastatic lesions of RCC, suggesting that some genetic changes are required for the primary tumor to metastasize, and many efforts are being made to identify them [35]. Based on these studies, various scoring systems have been proposed to predict the prognosis of patients but, unfortunately, there is still no standardized prognosis prediction system [36,37]. The histologic subtypes, sarcomatous differentiation, and nuclear grade that are found to be associated with the pattern of organotropic metastasis in this study are all information that is currently included in pathology reports. The findings of this study may assist clinicians in planning the follow-up of patients after complete resection of the primary cancer, or in deciding whether to perform additional lymphadenectomy.

## 5. Conclusions

This study confirmed the previously reported differences in metastatic behavior between histological subtypes of RCC. Additionally, prognostic factors such as nuclear grade and sarcomatous differentiation may affect metastatic behavior. The histological subtypes, nuclear grade, and sarcomatoid differentiation evaluated in this study are routine features in the histological evaluation of RCC and are usually described in pathology reports. Therefore, the findings of this study can be used by clinicians without additional steps, and we hope that this study will help provide better care for patients with kidney cancer.

## Figures and Tables

**Table 1:** Summary of the organotropic metastasis rates according to different renal cell carcinoma histologic subtypes.

	Clear Cell	Papillary	Chromophobe	Sarcomatoid
Metastasis Rate (Pts with metastasis/total number of Pts)				
Total	10.2% (8549/83,558)	4.6% (808/17,638)	1.9% (146/7847)	56.7% (673/1187)
Organotropic Metastasis Rate (Pts with metastasis to the indicated organ/Pts with metastasis)				
Bone	37.7% (3173/8417)	33.6% (267/795)	38.9% (56/144)	42.4% (281/662)
Brain	11.9% (996/8375)	6.1% (48/790)	6.9% (10/144)	11.0% (72/657)
Liver	15.3% (1281/8383)	18.8% (148/789)	27.6% (40/145)	23.9% (156/654)
Lung	61.6% (5156/8368)	46.6% (368/789)	41.0% (59/144)	66.0% (433/656)
LN	19.8% (846/4279)	37.2% (146/393)	17.5% (14/80)	21.9% (44/201)
Other	28.7% (1234/4298)	30.9% (121/391)	26.6% (21/79)	32.2% (65/202)

Pts, patients; LN, lymph node.

**Table 2:** Summary of the organotropic metastasis rates according to Fuhrman nuclear grade and World Health Organization/International Society of Urological Pathology (WHO/ISUP) nuclear grade in clear-cell renal cell carcinoma.

Fuhrman Grade	1	2	3	4
Metastasis Rate (Pts with metastasis/total number of Pts)				
	2.2% (126/5652)	3.5% (907/26,137)	11.0% (1478/13,495)	30.2% (1034/3420)
Organotropic Metastasis Rate (Pts with metastasis to the indicated organ/Pts with metastasis)				
Bone	38.2% (47/123)	34.9% (313/898)	31.8% (463/1457)	26.5% (272/1028)
Brain	11.4% (14/123)	10.7% (96/898)	10.1% (148/1459)	9.1% (93/1021)
Liver	11.3% (14/124)	12.9% (116/899)	12.4% (181/1455)	12.7% (130/1026)
Lung	54.1% (66/122)	54.7% (490/895)	61.0% (891/1460)	68.1% (694/1019)
LN	12.1% (4/33)	16.6% (40/241)	17.2% (65/377)	19.6% (64/326)
Other	27.3% (9/33)	28.0% (68/243)	27.5% (104/378)	22.8% (75/329)
WHO/ISUP grade	1	2	3	4
Metastasis Rate (Pts with metastasis/total number of Pts)				
	0.8% (19/2311)	1.3% (136/10,232)	5.9% (295/4967)	22.6% (416/1841)
Organotropic Metastasis Rate (Pts with metastasis to the indicated organ/Pts with metastasis)				
Bone	15.8% (3/19)	24.6% (33/134)	22.4% (66/295)	25.4% (105/413)
Brain	15.8% (3/19)	4.4% (6/135)	7.8% (23/295)	6.8% (28/413)
Liver	21.1% (4/19)	6.7% (9/135)	6.1% (18/295)	9.9% (41/414)
Lung	36.8% (7/19)	41.4% (55/133)	55.3% (163/295)	64.9% (268/413)
LN	5.3% (1/19)	8.9% (12/135)	11.9% (35/294)	17.2% (71/413)
Other	57.9% (11/19)	30.9% (42/136)	31.2% (92/295)	30.9% (128/414)

World Health Organization/International Society of Urological Pathology, WHO/ISUP; Pts, patients; LN, lymph node.

## Data Availability

The data presented in this study are available on request from the corresponding author.
